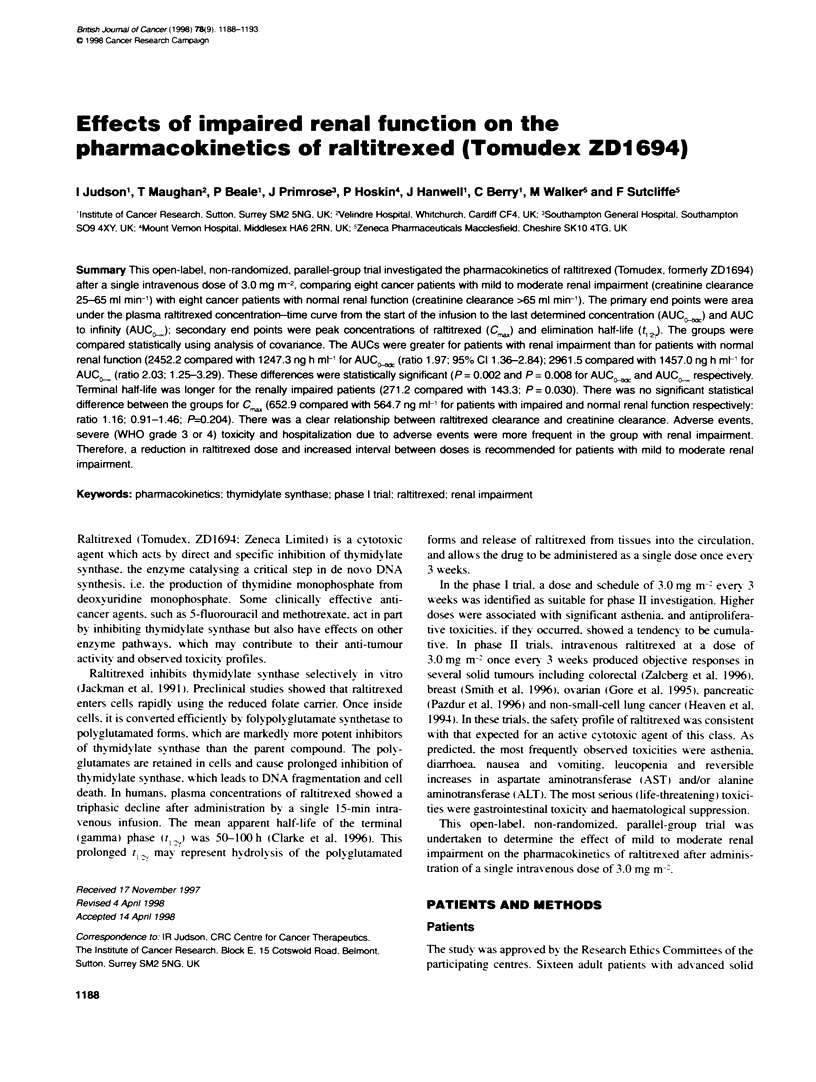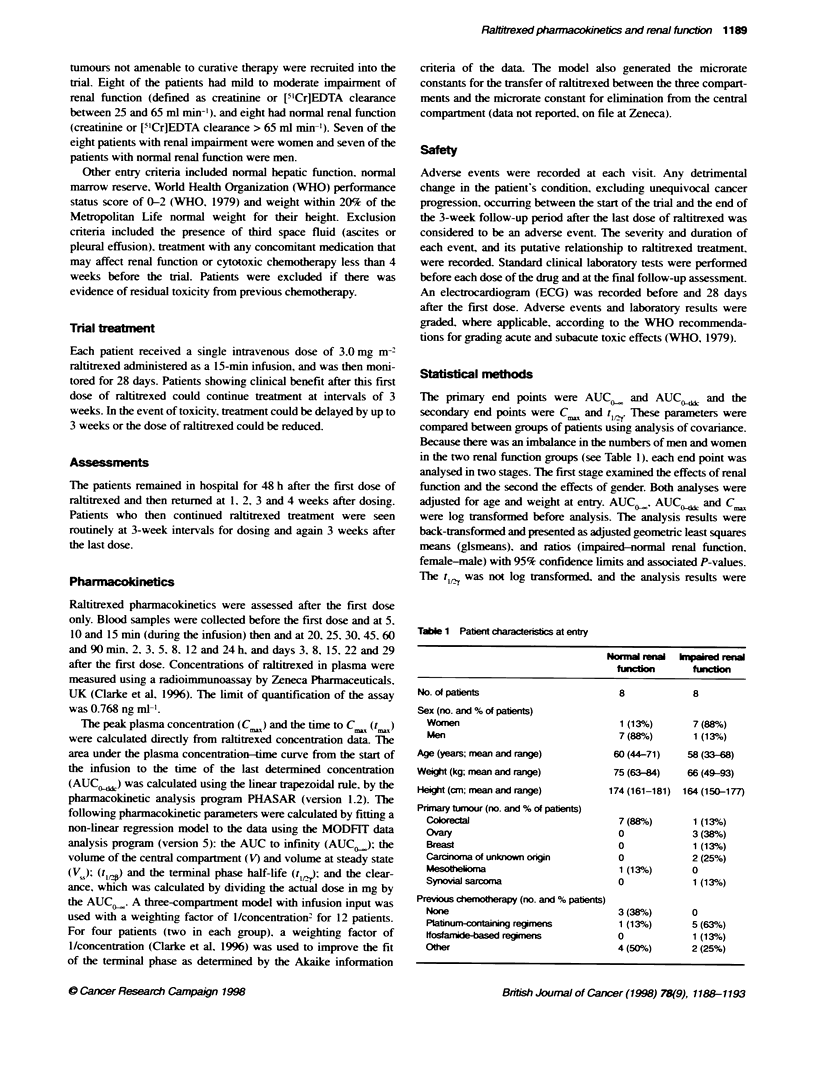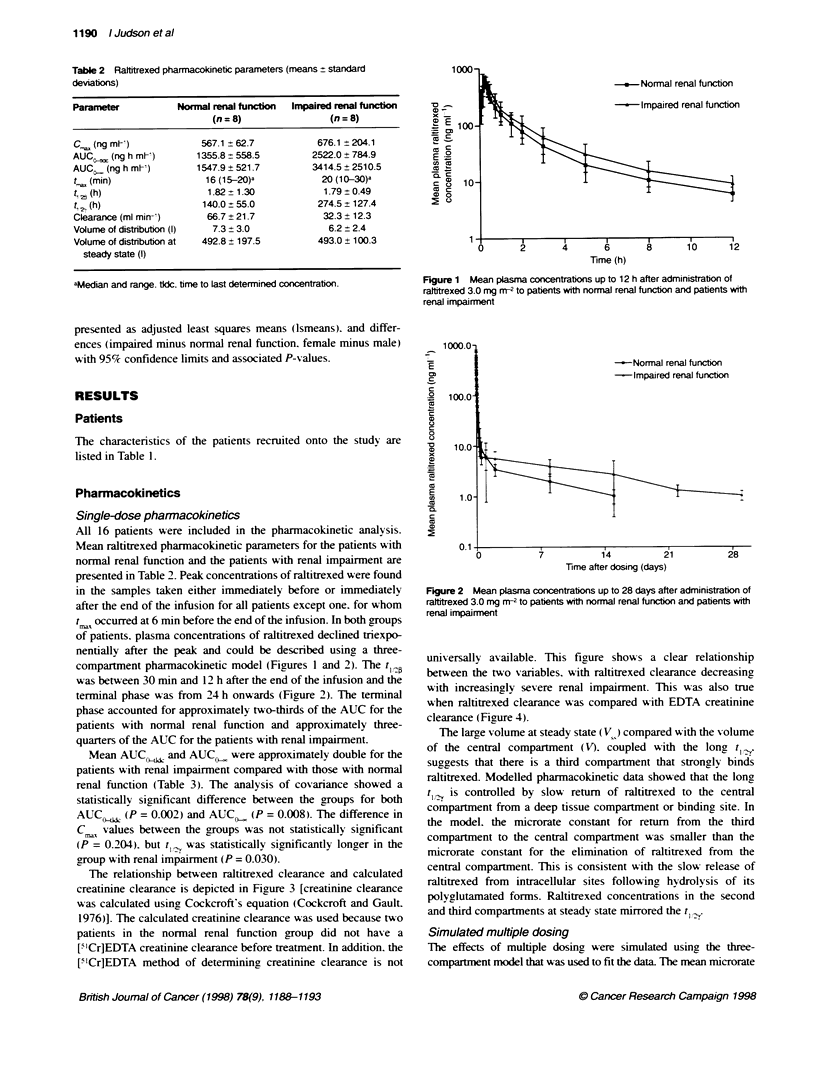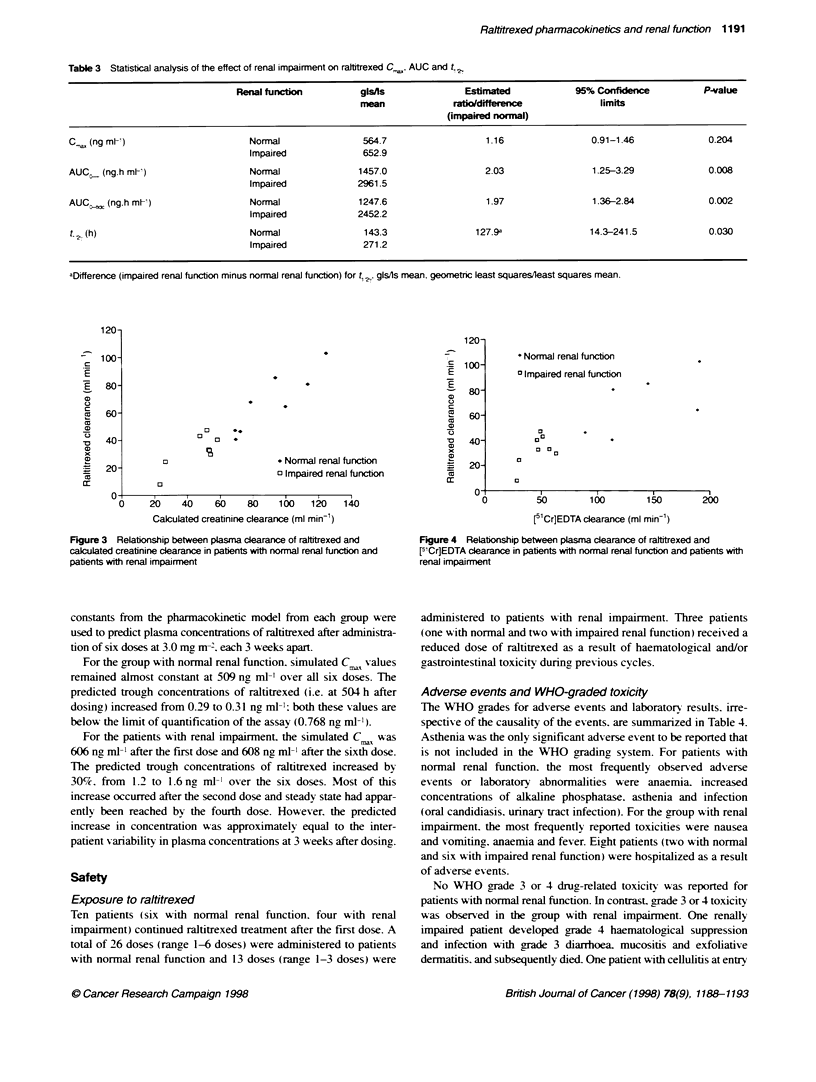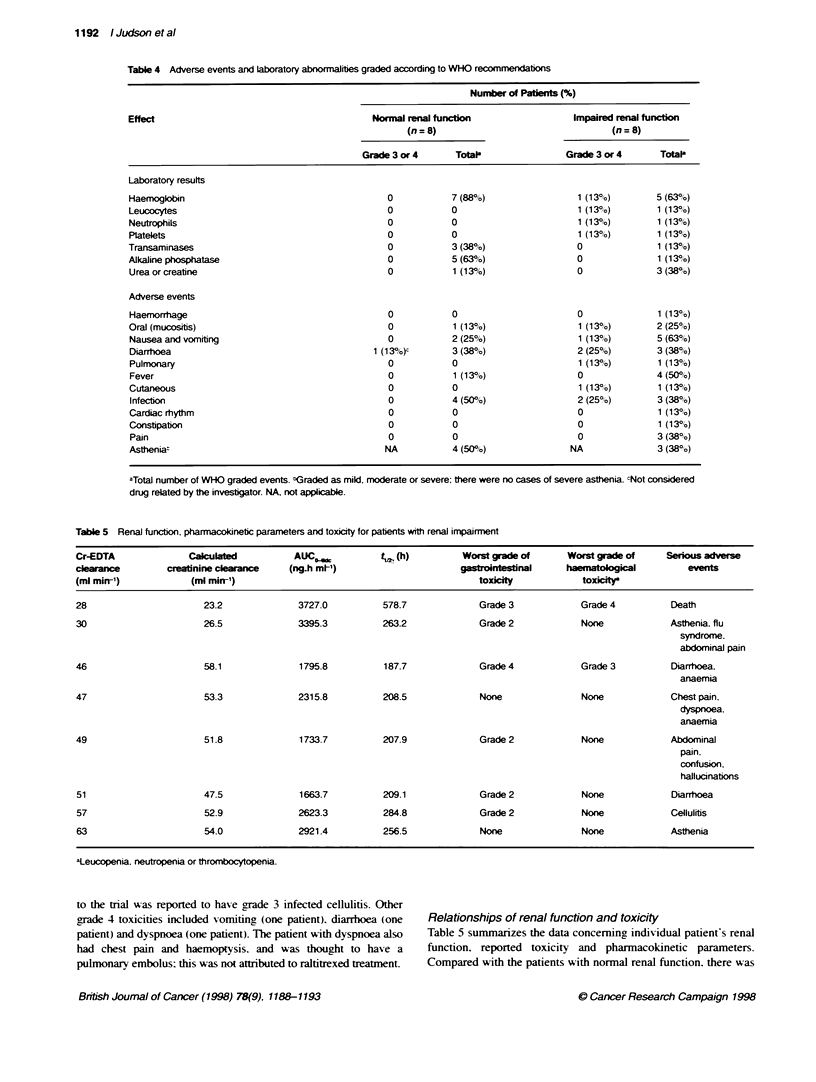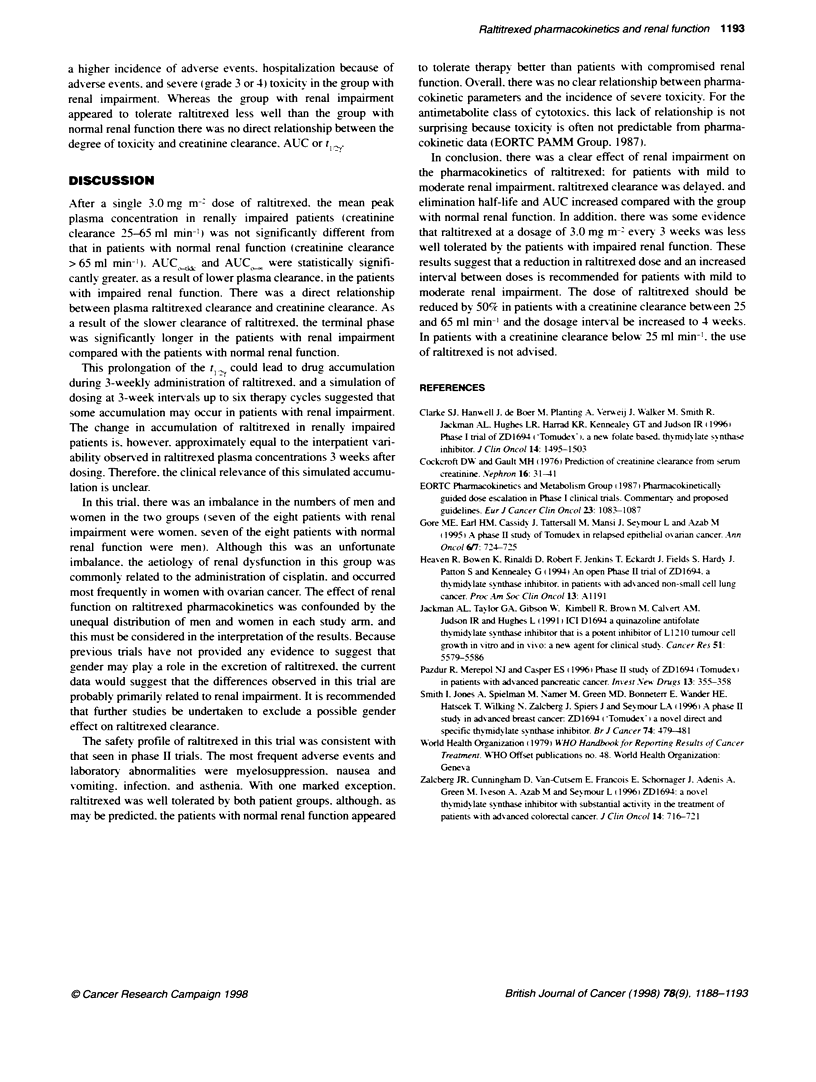# Effects of impaired renal function on the pharmacokinetics of raltitrexed (Tomudex ZD1694).

**DOI:** 10.1038/bjc.1998.652

**Published:** 1998-11

**Authors:** I. Judson, T. Maughan, P. Beale, J. Primrose, P. Hoskin, J. Hanwell, C. Berry, M. Walker, F. Sutcliffe

**Affiliations:** Institute of Cancer Research, Sutton, Surrey, UK.

## Abstract

This open-label, non-randomized, parallel-group trial investigated the pharmacokinetics of raltitrexed (Tomudex, formerly ZD1694) after a single intravenous dose of 3.0 mg m(-2), comparing eight cancer patients with mild to moderate renal impairment (creatinine clearance 25-65 ml min(-1)) with eight cancer patients with normal renal function (creatinine clearance >65 ml min(-1)). The primary end points were area under the plasma raltitrexed concentration-time curve from the start of the infusion to the last determined concentration (AUC(0-tldc)) and AUC to infinity (AUC(0-infinity)); secondary end points were peak concentrations of raltitrexed (Cmax) and elimination half-life (t(1/2gamma)). The groups were compared statistically using analysis of covariance. The AUCs were greater for patients with renal impairment than for patients with normal renal function (2452.2 compared with 1247.3 ng h ml(-1) for AUC(0-tldc) (ratio 1.97; 95% CI 1.36-2.84); 2961.5 compared with 1457.0 ng h ml(-1) for AUC(0-infinity) (ratio 2.03; 1.25-3.29). These differences were statistically significant (P = 0.002 and P = 0.008 for AUC(0-tldc) and AUC(0-infinity) respectively. Terminal half-life was longer for the renally impaired patients (271.2 compared with 143.3; P = 0.030). There was no significant statistical difference between the groups for Cmax (652.9 compared with 564.7 ng ml(-1) for patients with impaired and normal renal function respectively: ratio 1.16; 0.91-1.46; P = 0.204). There was a clear relationship between raltitrexed clearance and creatinine clearance. Adverse events, severe (WHO grade 3 or 4) toxicity and hospitalization due to adverse events were more frequent in the group with renal impairment. Therefore, a reduction in raltitrexed dose and increased interval between doses is recommended for patients with mild to moderate renal impairment.


British Journal of Cancer (1998) 78(9), 1188–1193
© 1998 Cancer Research Campaign

### Effects of impaired renal function on the pharmacokinetics of raltitrexed (Tomudex ZD1694)

I Judson[1], T Maughan[2], P Beale[1], J Primrose[3], P Hoskin[4], J Hanwell[1], C Berry[1], M Walker[5] and F Sutcliffe[5]

[1]Institute of Cancer Research, Sutton, Surrey SM2 5NG, UK; [2]Velindre Hospital, Whitchurch, Cardiff CF4, UK; [3]Southampton General Hospital, Southampton SO9 4XY, UK; [4]Mount Vernon Hospital, Middlesex HA6 2RN, UK; [5]Zeneca Pharmaceuticals Macclesfield, Cheshire SK10 4TG, UK

**Summary** This open-label, non-randomized, parallel-group trial investigated the pharmacokinetics of raltitrexed (Tomudex, formerly ZD1694) after a single intravenous dose of 3.0 mg $m^{-2}$, comparing eight cancer patients with mild to moderate renal impairment (creatinine clearance 25–65 ml $min^{-1}$) with eight cancer patients with normal renal function (creatinine clearance >65 ml $min^{-1}$). The primary end points were area under the plasma raltitrexed concentration–time curve from the start of the infusion to the last determined concentration ($AUC_{0-tldc}$) and AUC to infinity ($AUC_{0-\infty}$); secondary end points were peak concentrations of raltitrexed ($C_{max}$) and elimination half-life ($t_{1/2\gamma}$). The groups were compared statistically using analysis of covariance. The AUCs were greater for patients with renal impairment than for patients with normal renal function (2452.2 compared with 1247.3 ng h $ml^{-1}$ for $AUC_{0-tldc}$ (ratio 1.97; 95% CI 1.36–2.84); 2961.5 compared with 1457.0 ng h $ml^{-1}$ for $AUC_{0-\infty}$ (ratio 2.03; 1.25–3.29). These differences were statistically significant ($P = 0.002$ and $P = 0.008$ for $AUC_{0-tldc}$ and $AUC_{0-\infty}$ respectively. Terminal half-life was longer for the renally impaired patients (271.2 compared with 143.3; $P = 0.030$). There was no significant statistical difference between the groups for $C_{max}$ (652.9 compared with 564.7 ng $ml^{-1}$ for patients with impaired and normal renal function respectively: ratio 1.16; 0.91–1.46; $P$=0.204). There was a clear relationship between raltitrexed clearance and creatinine clearance. Adverse events, severe (WHO grade 3 or 4) toxicity and hospitalization due to adverse events were more frequent in the group with renal impairment. Therefore, a reduction in raltitrexed dose and increased interval between doses is recommended for patients with mild to moderate renal impairment.

**Keywords:** pharmacokinetics; thymidylate synthase; phase I trial; raltitrexed; renal impairment

Raltitrexed (Tomudex, ZD1694; Zeneca Limited) is a cytotoxic agent which acts by direct and specific inhibition of thymidylate synthase, the enzyme catalysing a critical step in de novo DNA synthesis, i.e. the production of thymidine monophosphate from deoxyuridine monophosphate. Some clinically effective anticancer agents, such as 5-fluorouracil and methotrexate, act in part by inhibiting thymidylate synthase but also have effects on other enzyme pathways, which may contribute to their anti-tumour activity and observed toxicity profiles.

Raltitrexed inhibits thymidylate synthase selectively in vitro (Jackman et al, 1991). Preclinical studies showed that raltitrexed enters cells rapidly using the reduced folate carrier. Once inside cells, it is converted efficiently by folypolyglutamate synthetase to polyglutamated forms, which are markedly more potent inhibitors of thymidylate synthase than the parent compound. The polyglutamates are retained in cells and cause prolonged inhibition of thymidylate synthase, which leads to DNA fragmentation and cell death. In humans, plasma concentrations of raltitrexed showed a triphasic decline after administration by a single 15-min intravenous infusion. The mean apparent half-life of the terminal (gamma) phase ($t_{1/2\gamma}$) was 50–100 h (Clarke et al, 1996). This prolonged $t_{1/2\gamma}$ may represent hydrolysis of the polyglutamated forms and release of raltitrexed from tissues into the circulation, and allows the drug to be administered as a single dose once every 3 weeks.

In the phase I trial, a dose and schedule of 3.0 mg $m^{-2}$ every 3 weeks was identified as suitable for phase II investigation. Higher doses were associated with significant asthenia, and antiproliferative toxicities, if they occurred, showed a tendency to be cumulative. In phase II trials, intravenous raltitrexed at a dose of 3.0 mg $m^{-2}$ once every 3 weeks produced objective responses in several solid tumours including colorectal (Zalcberg et al, 1996), breast (Smith et al, 1996), ovarian (Gore et al, 1995), pancreatic (Pazdur et al, 1996) and non-small-cell lung cancer (Heaven et al, 1994). In these trials, the safety profile of raltitrexed was consistent with that expected for an active cytotoxic agent of this class. As predicted, the most frequently observed toxicities were asthenia, diarrhoea, nausea and vomiting, leucopenia and reversible increases in aspartate aminotransferase (AST) and/or alanine aminotransferase (ALT). The most serious (life-threatening) toxicities were gastrointestinal toxicity and haematological suppression.

This open-label, non-randomized, parallel-group trial was undertaken to determine the effect of mild to moderate renal impairment on the pharmacokinetics of raltitrexed after administration of a single intravenous dose of 3.0 mg $m^{-2}$.

*Received 17 November 1997*

*Revised 4 April 1998*

*Accepted 14 April 1998*

*Correspondence to:* IR Judson, CRC Centre for Cancer Therapeutics, The Institute of Cancer Research, Block E, 15 Cotswold Road, Belmont, Sutton, Surrey SM2 5NG, UK

### PATIENTS AND METHODS

### Patients

The study was approved by the Research Ethics Committees of the participating centres. Sixteen adult patients with advanced solid tumours not amenable to curative therapy were recruited into the trial. Eight of the patients had mild to moderate impairment of renal function (defined as creatinine or [$^{51}$Cr]EDTA clearance between 25 and 65 ml min$^{-1}$), and eight had normal renal function (creatinine or [$^{51}$Cr]EDTA clearance > 65 ml min$^{-1}$). Seven of the eight patients with renal impairment were women and seven of the patients with normal renal function were men.

Other entry criteria included normal hepatic function, normal marrow reserve, World Health Organization (WHO) performance status score of 0–2 (WHO, 1979) and weight within 20% of the Metropolitan Life normal weight for their height. Exclusion criteria included the presence of third space fluid (ascites or pleural effusion), treatment with any concomitant medication that may affect renal function or cytotoxic chemotherapy less than 4 weeks before the trial. Patients were excluded if there was evidence of residual toxicity from previous chemotherapy.

### Trial treatment

Each patient received a single intravenous dose of 3.0 mg m$^{-2}$ raltitrexed administered as a 15-min infusion, and was then monitored for 28 days. Patients showing clinical benefit after this first dose of raltitrexed could continue treatment at intervals of 3 weeks. In the event of toxicity, treatment could be delayed by up to 3 weeks or the dose of raltitrexed could be reduced.

### Assessments

The patients remained in hospital for 48 h after the first dose of raltitrexed and then returned at 1, 2, 3 and 4 weeks after dosing. Patients who then continued raltitrexed treatment were seen routinely at 3-week intervals for dosing and again 3 weeks after the last dose.

### Pharmacokinetics

Raltitrexed pharmacokinetics were assessed after the first dose only. Blood samples were collected before the first dose and at 5, 10 and 15 min (during the infusion) then and at 20, 25, 30, 45, 60 and 90 min, 2, 3, 5, 8, 12 and 24 h, and days 3, 8, 15, 22 and 29 after the first dose. Concentrations of raltitrexed in plasma were measured using a radioimmunoassay by Zeneca Pharmaceuticals, UK (Clarke et al, 1996). The limit of quantification of the assay was 0.768 ng ml$^{-1}$.

The peak plasma concentration ($C_{max}$) and the time to $C_{max}$ ($t_{max}$) were calculated directly from raltitrexed concentration data. The area under the plasma concentration–time curve from the start of the infusion to the time of the last determined concentration ($AUC_{0-tldc}$) was calculated using the linear trapezoidal rule, by the pharmacokinetic analysis program PHASAR (version 1.2). The following pharmacokinetic parameters were calculated by fitting a non-linear regression model to the data using the MODFIT data analysis program (version 5): the AUC to infinity ($AUC_{0-\infty}$); the volume of the central compartment ($V$) and volume at steady state ($V_{ss}$); ($t_{1/2\beta}$) and the terminal phase half-life ($t_{1/2\gamma}$); and the clearance, which was calculated by dividing the actual dose in mg by the $AUC_{0-\infty}$. A three-compartment model with infusion input was used with a weighting factor of 1/concentration$^2$ for 12 patients. For four patients (two in each group), a weighting factor of 1/concentration (Clarke et al, 1996) was used to improve the fit of the terminal phase as determined by the Akaike information criteria of the data. The model also generated the microrate constants for the transfer of raltitrexed between the three compartments and the microrate constant for elimination from the central compartment (data not reported, on file at Zeneca).

### Safety

Adverse events were recorded at each visit. Any detrimental change in the patient's condition, excluding unequivocal cancer progression, occurring between the start of the trial and the end of the 3-week follow-up period after the last dose of raltitrexed was considered to be an adverse event. The severity and duration of each event, and its putative relationship to raltitrexed treatment, were recorded. Standard clinical laboratory tests were performed before each dose of the drug and at the final follow-up assessment. An electrocardiogram (ECG) was recorded before and 28 days after the first dose. Adverse events and laboratory results were graded, where applicable, according to the WHO recommendations for grading acute and subacute toxic effects (WHO, 1979).

### Statistical methods

The primary end points were $AUC_{0-\infty}$ and $AUC_{0-tldc}$ and the secondary end points were $C_{max}$ and $t_{1/2\gamma}$. These parameters were compared between groups of patients using analysis of covariance. Because there was an imbalance in the numbers of men and women in the two renal function groups (see Table 1), each end point was analysed in two stages. The first stage examined the effects of renal function and the second the effects of gender. Both analyses were adjusted for age and weight at entry. $AUC_{0-\infty}$, $AUC_{0-tldc}$ and $C_{max}$ were log transformed before analysis. The analysis results were back-transformed and presented as adjusted geometric least squares means (glsmeans), and ratios (impaired–normal renal function, female–male) with 95% confidence limits and associated $P$-values. The $t_{1/2\gamma}$ was not log transformed, and the analysis results were

**Table 1** Patient characteristics at entry

| | Normal renal function | Impaired renal function |
|---|---|---|
| No. of patients | 8 | 8 |
| Sex (no. and % of patients) | | |
| Women | 1 (13%) | 7 (88%) |
| Men | 7 (88%) | 1 (13%) |
| Age (years; mean and range) | 60 (44–71) | 58 (33–68) |
| Weight (kg; mean and range) | 75 (63–84) | 66 (49–93) |
| Height (cm; mean and range) | 174 (161–181) | 164 (150–177) |
| Primary tumour (no. and % of patients) | | |
| Colorectal | 7 (88%) | 1 (13%) |
| Ovary | 0 | 3 (38%) |
| Breast | 0 | 1 (13%) |
| Carcinoma of unknown origin | 0 | 2 (25%) |
| Mesothelioma | 1 (13%) | 0 |
| Synovial sarcoma | 0 | 1 (13%) |
| Previous chemotherapy (no. and % patients) | | |
| None | 3 (38%) | 0 |
| Platinum-containing regimens | 1 (13%) | 5 (63%) |
| Ifosfamide-based regimens | 0 | 1 (13%) |
| Other | 4 (50%) | 2 (25%) |

© Cancer Research Campaign 1998

Table 2 Raltitrexed pharmacokinetic parameters (means ± standard deviations)

| Parameter | Normal renal function ($n$ = 8) | Impaired renal function ($n$ = 8) |
|---|---|---|
| $C_{max}$ (ng ml$^{-1}$) | 567.1 ± 62.7 | 676.1 ± 204.1 |
| $AUC_{0-tldc}$ (ng h ml$^{-1}$) | 1355.8 ± 558.5 | 2522.0 ± 784.9 |
| $AUC_{0-\infty}$ (ng h ml$^{-1}$) | 1547.9 ± 521.7 | 3414.5 ± 2510.5 |
| $t_{max}$ (min) | 16 (15–20)[a] | 20 (10–30)[a] |
| $t_{1/2\beta}$ (h) | 1.82 ± 1.30 | 1.79 ± 0.49 |
| $t_{1/2\gamma}$ (h) | 140.0 ± 55.0 | 274.5 ± 127.4 |
| Clearance (ml min$^{-1}$) | 66.7 ± 21.7 | 32.3 ± 12.3 |
| Volume of distribution (l) | 7.3 ± 3.0 | 6.2 ± 2.4 |
| Volume of distribution at steady state (l) | 492.8 ± 197.5 | 493.0 ± 100.3 |

[a]Median and range. tldc, time to last determined concentration.

presented as adjusted least squares means (lsmeans), and differences (impaired minus normal renal function, female minus male) with 95% confidence limits and associated $P$-values.

### RESULTS

### Patients

The characteristics of the patients recruited onto the study are listed in Table 1.

### Pharmacokinetics

### *Single-dose pharmacokinetics*

All 16 patients were included in the pharmacokinetic analysis. Mean raltitrexed pharmacokinetic parameters for the patients with normal renal function and the patients with renal impairment are presented in Table 2. Peak concentrations of raltitrexed were found in the samples taken either immediately before or immediately after the end of the infusion for all patients except one, for whom $t_{max}$ occurred at 6 min before the end of the infusion. In both groups of patients, plasma concentrations of raltitrexed declined triexponentially after the peak and could be described using a three-compartment pharmacokinetic model (Figures 1 and 2). The $t_{1/2\beta}$ was between 30 min and 12 h after the end of the infusion and the terminal phase was from 24 h onwards (Figure 2). The terminal phase accounted for approximately two-thirds of the AUC for the patients with normal renal function and approximately three-quarters of the AUC for the patients with renal impairment.

Mean $AUC_{0-tldc}$ and $AUC_{0-\infty}$ were approximately double for the patients with renal impairment compared with those with normal renal function (Table 3). The analysis of covariance showed a statistically significant difference between the groups for both $AUC_{0-tldc}$ ($P$ = 0.002) and $AUC_{0-\infty}$ ($P$ = 0.008). The difference in $C_{max}$ values between the groups was not statistically significant ($P$ = 0.204), but $t_{1/2\gamma}$ was statistically significantly longer in the group with renal impairment ($P$ = 0.030).

The relationship between raltitrexed clearance and calculated creatinine clearance is depicted in Figure 3 [creatinine clearance was calculated using Cockcroft's equation (Cockcroft and Gault, 1976)]. The calculated creatinine clearance was used because two patients in the normal renal function group did not have a [$^{51}$Cr]EDTA creatinine clearance before treatment. In addition, the [$^{51}$Cr]EDTA method of determining creatinine clearance is not

Figure 1 Mean plasma concentrations up to 12 h after administration of raltitrexed 3.0 mg m$^{-2}$ to patients with normal renal function and patients with renal impairment

Figure 2 Mean plasma concentrations up to 28 days after administration of raltitrexed 3.0 mg m$^{-2}$ to patients with normal renal function and patients with renal impairment universally available. This figure shows a clear relationship between the two variables, with raltitrexed clearance decreasing with increasingly severe renal impairment. This was also true when raltitrexed clearance was compared with EDTA creatinine clearance (Figure 4).

The large volume at steady state ($V_{ss}$) compared with the volume of the central compartment ($V$), coupled with the long $t_{1/2\gamma}$, suggests that there is a third compartment that strongly binds raltitrexed. Modelled pharmacokinetic data showed that the long $t_{1/2\gamma}$ is controlled by slow return of raltitrexed to the central compartment from a deep tissue compartment or binding site. In the model, the microrate constant for return from the third compartment to the central compartment was smaller than the microrate constant for the elimination of raltitrexed from the central compartment. This is consistent with the slow release of raltitrexed from intracellular sites following hydrolysis of its polyglutamated forms. Raltitrexed concentrations in the second and third compartments at steady state mirrored the $t_{1/2\gamma}$.

### *Simulated multiple dosing*

The effects of multiple dosing were simulated using the three-compartment model that was used to fit the data. The mean microrate

© Cancer Research Campaign 1998

**Table 3** Statistical analysis of the effect of renal impairment on raltitrexed $C_{max}$, AUC and $t_{1/2\gamma}$

| | Renal function | gls/ls mean | Estimated ratio/difference (impaired normal) | 95% Confidence limits | *P*-value |
|---|---|---|---|---|---|
| $C_{max}$ (ng ml$^{-1}$) | Normal | 564.7 | 1.16 | 0.91–1.46 | 0.204 |
| | Impaired | 652.9 | | | |
| $AUC_{0-\infty}$ (ng.h ml$^{-1}$) | Normal | 1457.0 | 2.03 | 1.25–3.29 | 0.008 |
| | Impaired | 2961.5 | | | |
| $AUC_{0-tlqc}$ (ng.h ml$^{-1}$) | Normal | 1247.6 | 1.97 | 1.36–2.84 | 0.002 |
| | Impaired | 2452.2 | | | |
| $t_{1/2\gamma}$ (h) | Normal | 143.3 | 127.9[a] | 14.3–241.5 | 0.030 |
| | Impaired | 271.2 | | | |

[a]Difference (impaired renal function minus normal renal function) for $t_{1/2\gamma}$. gls/ls mean, geometric least squares/least squares mean.

**Figure 3** Relationship between plasma clearance of raltitrexed and calculated creatinine clearance in patients with normal renal function and patients with renal impairment

**Figure 4** Relationship between plasma clearance of raltitrexed and [$^{51}$Cr]EDTA clearance in patients with normal renal function and patients with renal impairment constants from the pharmacokinetic model from each group were used to predict plasma concentrations of raltitrexed after administration of six doses at 3.0 mg m$^{-2}$, each 3 weeks apart.

For the group with normal renal function, simulated $C_{max}$ values remained almost constant at 509 ng ml$^{-1}$ over all six doses. The predicted trough concentrations of raltitrexed (i.e. at 504 h after dosing) increased from 0.29 to 0.31 ng ml$^{-1}$: both these values are below the limit of quantification of the assay (0.768 ng ml$^{-1}$).

For the patients with renal impairment, the simulated $C_{max}$ was 606 ng ml$^{-1}$ after the first dose and 608 ng ml$^{-1}$ after the sixth dose. The predicted trough concentrations of raltitrexed increased by 30%, from 1.2 to 1.6 ng ml$^{-1}$ over the six doses. Most of this increase occurred after the second dose and steady state had apparently been reached by the fourth dose. However, the predicted increase in concentration was approximately equal to the interpatient variability in plasma concentrations at 3 weeks after dosing.

### Safety

### *Exposure to raltitrexed*

Ten patients (six with normal renal function, four with renal impairment) continued raltitrexed treatment after the first dose. A total of 26 doses (range 1–6 doses) were administered to patients with normal renal function and 13 doses (range 1–3 doses) were administered to patients with renal impairment. Three patients (one with normal and two with impaired renal function) received a reduced dose of raltitrexed as a result of haematological and/or gastrointestinal toxicity during previous cycles.

### *Adverse events and WHO-graded toxicity*

The WHO grades for adverse events and laboratory results, irrespective of the causality of the events, are summarized in Table 4. Asthenia was the only significant adverse event to be reported that is not included in the WHO grading system. For patients with normal renal function, the most frequently observed adverse events or laboratory abnormalities were anaemia, increased concentrations of alkaline phosphatase, asthenia and infection (oral candidiasis, urinary tract infection). For the group with renal impairment, the most frequently reported toxicities were nausea and vomiting, anaemia and fever. Eight patients (two with normal and six with impaired renal function) were hospitalized as a result of adverse events.

No WHO grade 3 or 4 drug-related toxicity was reported for patients with normal renal function. In contrast, grade 3 or 4 toxicity was observed in the group with renal impairment. One renally impaired patient developed grade 4 haematological suppression and infection with grade 3 diarrhoea, mucositis and exfoliative dermatitis, and subsequently died. One patient with cellulitis at entry

**Table 4** Adverse events and laboratory abnormalities graded according to WHO recommendations

| Effect | Number of Patients (%) | | | |
|---|---|---|---|---|
| | Normal renal function (n = 8) | | Impaired renal function (n = 8) | |
| | Grade 3 or 4 | Total[a] | Grade 3 or 4 | Total[a] |
| Laboratory results | | | | |
| Haemoglobin | 0 | 7 (88%) | 1 (13%) | 5 (63%) |
| Leucocytes | 0 | 0 | 1 (13%) | 1 (13%) |
| Neutrophils | 0 | 0 | 1 (13%) | 1 (13%) |
| Platelets | 0 | 0 | 1 (13%) | 1 (13%) |
| Transaminases | 0 | 3 (38%) | 0 | 1 (13%) |
| Alkaline phosphatase | 0 | 5 (63%) | 0 | 1 (13%) |
| Urea or creatine | 0 | 1 (13%) | 0 | 3 (38%) |
| Adverse events | | | | |
| Haemorrhage | 0 | 0 | 0 | 1 (13%) |
| Oral (mucositis) | 0 | 1 (13%) | 1 (13%) | 2 (25%) |
| Nausea and vomiting | 0 | 2 (25%) | 1 (13%) | 5 (63%) |
| Diarrhoea | 1 (13%)[c] | 3 (38%) | 2 (25%) | 3 (38%) |
| Pulmonary | 0 | 0 | 1 (13%) | 1 (13%) |
| Fever | 0 | 1 (13%) | 0 | 4 (50%) |
| Cutaneous | 0 | 0 | 1 (13%) | 1 (13%) |
| Infection | 0 | 4 (50%) | 2 (25%) | 3 (38%) |
| Cardiac rhythm | 0 | 0 | 0 | 1 (13%) |
| Constipation | 0 | 0 | 0 | 1 (13%) |
| Pain | 0 | 0 | 0 | 3 (38%) |
| Asthenia[b] | NA | 4 (50%) | NA | 3 (38%) |

[a]Total number of WHO graded events. [b]Graded as mild, moderate or severe; there were no cases of severe asthenia. [c]Not considered drug related by the investigator. NA, not applicable.

**Table 5** Renal function, pharmacokinetic parameters and toxicity for patients with renal impairment

| Cr-EDTA clearance (ml min$^{-1}$) | Calculated creatinine clearance (ml min$^{-1}$) | $AUC_{0-tldc}$ (ng.h ml$^{-1}$) | $t_{1/2\gamma}$ (h) | Worst grade of gastrointestinal toxicity | Worst grade of haematological toxicity[a] | Serious adverse events |
|---|---|---|---|---|---|---|
| 28 | 23.2 | 3727.0 | 578.7 | Grade 3 | Grade 4 | Death |
| 30 | 26.5 | 3395.3 | 263.2 | Grade 2 | None | Asthenia, flu syndrome, abdominal pain |
| 46 | 58.1 | 1795.8 | 187.7 | Grade 4 | Grade 3 | Diarrhoea, anaemia |
| 47 | 53.3 | 2315.8 | 208.5 | None | None | Chest pain, dyspnoea, anaemia |
| 49 | 51.8 | 1733.7 | 207.9 | Grade 2 | None | Abdominal pain, confusion, hallucinations |
| 51 | 47.5 | 1663.7 | 209.1 | Grade 2 | None | Diarrhoea |
| 57 | 52.9 | 2623.3 | 284.8 | Grade 2 | None | Cellulitis |
| 63 | 54.0 | 2921.4 | 256.5 | None | None | Asthenia |

[a]Leucopenia, neutropenia or thrombocytopenia.

to the trial was reported to have grade 3 infected cellulitis. Other grade 4 toxicities included vomiting (one patient), diarrhoea (one patient) and dyspnoea (one patient). The patient with dyspnoea also had chest pain and haemoptysis, and was thought to have a pulmonary embolus; this was not attributed to raltitrexed treatment.

### *Relationships of renal function and toxicity*

Table 5 summarizes the data concerning individual patient's renal function, reported toxicity and pharmacokinetic parameters. Compared with the patients with normal renal function, there was a higher incidence of adverse events, hospitalization because of adverse events, and severe (grade 3 or 4) toxicity in the group with renal impairment. Whereas the group with renal impairment appeared to tolerate raltitrexed less well than the group with normal renal function there was no direct relationship between the degree of toxicity and creatinine clearance, AUC or $t_{1/2\gamma}$.

### DISCUSSION

After a single 3.0 mg $m^{-2}$ dose of raltitrexed, the mean peak plasma concentration in renally impaired patients (creatinine clearance 25–65 ml $min^{-1}$) was not significantly different from that in patients with normal renal function (creatinine clearance > 65 ml $min^{-1}$). $AUC_{0-tldc}$ and $AUC_{0-\infty}$ were statistically significantly greater, as a result of lower plasma clearance, in the patients with impaired renal function. There was a direct relationship between plasma raltitrexed clearance and creatinine clearance. As a result of the slower clearance of raltitrexed, the terminal phase was significantly longer in the patients with renal impairment compared with the patients with normal renal function.

This prolongation of the $t_{1/2\gamma}$ could lead to drug accumulation during 3-weekly administration of raltitrexed, and a simulation of dosing at 3-week intervals up to six therapy cycles suggested that some accumulation may occur in patients with renal impairment. The change in accumulation of raltitrexed in renally impaired patients is, however, approximately equal to the interpatient variability observed in raltitrexed plasma concentrations 3 weeks after dosing. Therefore, the clinical relevance of this simulated accumulation is unclear.

In this trial, there was an imbalance in the numbers of men and women in the two groups (seven of the eight patients with renal impairment were women, seven of the eight patients with normal renal function were men). Although this was an unfortunate imbalance, the aetiology of renal dysfunction in this group was commonly related to the administration of cisplatin, and occurred most frequently in women with ovarian cancer. The effect of renal function on raltitrexed pharmacokinetics was confounded by the unequal distribution of men and women in each study arm, and this must be considered in the interpretation of the results. Because previous trials have not provided any evidence to suggest that gender may play a role in the excretion of raltitrexed, the current data would suggest that the differences observed in this trial are probably primarily related to renal impairment. It is recommended that further studies be undertaken to exclude a possible gender effect on raltitrexed clearance.

The safety profile of raltitrexed in this trial was consistent with that seen in phase II trials. The most frequent adverse events and laboratory abnormalities were myelosuppression, nausea and vomiting, infection, and asthenia. With one marked exception, raltitrexed was well tolerated by both patient groups, although, as may be predicted, the patients with normal renal function appeared to tolerate therapy better than patients with compromised renal function. Overall, there was no clear relationship between pharmacokinetic parameters and the incidence of severe toxicity. For the antimetabolite class of cytotoxics, this lack of relationship is not surprising because toxicity is often not predictable from pharmacokinetic data (EORTC PAMM Group, 1987).

In conclusion, there was a clear effect of renal impairment on the pharmacokinetics of raltitrexed: for patients with mild to moderate renal impairment, raltitrexed clearance was delayed, and elimination half-life and AUC increased compared with the group with normal renal function. In addition, there was some evidence that raltitrexed at a dosage of 3.0 mg $m^{-2}$ every 3 weeks was less well tolerated by the patients with impaired renal function. These results suggest that a reduction in raltitrexed dose and an increased interval between doses is recommended for patients with mild to moderate renal impairment. The dose of raltitrexed should be reduced by 50% in patients with a creatinine clearance between 25 and 65 ml $min^{-1}$ and the dosage interval be increased to 4 weeks. In patients with a creatinine clearance below 25 ml $min^{-1}$, the use of raltitrexed is not advised.

### REFERENCES

Clarke SJ, Hanwell J, de Boer M, Planting A, Verweij J, Walker M, Smith R, Jackman AL, Hughes LR, Harrad KR, Kennealey GT and Judson IR (1996) Phase I trial of ZD1694 ('Tomudex'), a new folate based, thymidylate synthase inhibitor. *J Clin Oncol* **14**: 1495–1503

Cockcroft DW and Gault MH (1976) Prediction of creatinine clearance from serum creatinine. *Nephron* **16**: 31–41

EORTC Pharmacokinetics and Metabolism Group (1987) Pharmacokinetically guided dose escalation in Phase I clinical trials. Commentary and proposed guidelines. *Eur J Cancer Clin Oncol* **23**: 1083–1087

Gore ME, Earl HM, Cassidy J, Tattersall M, Mansi J, Seymour L and Azab M (1995) A phase II study of Tomudex in relapsed epithelial ovarian cancer. *Ann Oncol* **6/7**: 724–725

Heaven R, Bowen K, Rinaldi D, Robert F, Jenkins T, Eckardt J, Fields S, Hardy J, Patton S and Kennealey G (1994) An open Phase II trial of ZD1694, a thymidylate synthase inhibitor, in patients with advanced non-small cell lung cancer. *Proc Am Soc Clin Oncol* **13**: A1191

Jackman AL, Taylor GA, Gibson W, Kimbell R, Brown M, Calvert AM, Judson IR and Hughes L (1991) ICI D1694 a quinazoline antifolate thymidylate synthase inhibitor that is a potent inhibitor of L1210 tumour cell growth in vitro and in vivo: a new agent for clinical study. *Cancer Res* **51**: 5579–5586

Pazdur R, Merepol NJ and Casper ES (1996) Phase II study of ZD1694 (Tomudex) in patients with advanced pancreatic cancer. *Invest New Drugs* **13**: 355–358

Smith I, Jones A, Spielman M, Namer M, Green MD, Bonneterr E, Wander HE, Hatscek T, Wilking N, Zalcberg J, Spiers J and Seymour LA (1996) A phase II study in advanced breast cancer: ZD1694 ('Tomudex') a novel direct and specific thymidylate synthase inhibitor. *Br J Cancer* **74**: 479–481

World Health Organization (1979) *WHO Handbook for Reporting Results of Cancer Treatment*. WHO Offset publications no. 48. World Health Organization: Geneva

Zalcberg JR, Cunningham D, Van-Cutsem E, Francois E, Schornager J, Adenis A, Green M, Iveson A, Azab M and Seymour L (1996) ZD1694: a novel thymidylate synthase inhibitor with substantial activity in the treatment of patients with advanced colorectal cancer. *J Clin Oncol* **14**: 716–721

© Cancer Research Campaign 1998